# Triticum monococcum lines with distinct metabolic phenotypes and phloem‐based partial resistance to the bird cherry–oat aphid Rhopalosiphum padi

**DOI:** 10.1111/aab.12274

**Published:** 2016-02-29

**Authors:** A.F.C. Greenslade, J.L. Ward, J.L. Martin, D.I. Corol, S.J. Clark, L.E. Smart, G.I. Aradottir

**Affiliations:** ^1^ Department of Biological Chemistry and Crop ProtectionRothamsted ResearchHertfordshireAL5 5JQUK; ^2^ Department of Plant Biology and Crop ScienceRothamsted ResearchHertfordshireAL5 5JQUK; ^3^ Department of Computational and Systems BiologyRothamsted ResearchHertfordshireAL5 2JQUK

**Keywords:** Aphid, crop protection, EPG, phloem feeding, resistance, Rhopalosiphum padi, Triticum monococcum, wheat

## Abstract

Crop protection is an integral part of establishing food security, by protecting the yield potential of crops. Cereal aphids cause yield losses by direct damage and transmission of viruses. Some wild relatives of wheat show resistance to aphids but the mechanisms remain unresolved. In order to elucidate the location of the partial resistance to the bird cherry–oat aphid, Rhopalosiphum padi, in diploid wheat lines of Triticum monococcum, we conducted aphid performance studies using developmental bioassays and electrical penetration graphs, as well as metabolic profiling of partially resistant and susceptible lines. This demonstrated that the partial resistance is related to a delayed effect on the reproduction and development of R. padi. The observed partial resistance is phloem based and is shown by an increase in number of probes before the first phloem ingestion, a higher number and duration of salivation events without subsequent phloem feeding and a shorter time spent phloem feeding on plants with reduced susceptibility. Clear metabolic phenotypes separate partially resistant and susceptible lines, with the former having lower levels of the majority of primary metabolites, including total carbohydrates. A number of compounds were identified as being at different levels in the susceptible and partially resistant lines, with asparagine, octopamine and glycine betaine elevated in less susceptible lines without aphid infestation. In addition, two of those, asparagine and octopamine, as well as threonine, glutamine, succinate, trehalose, glycerol, guanosine and choline increased in response to infestation, accumulating in plant tissue localised close to aphid feeding after 24 h. There was no clear evidence of systemic plant response to aphid infestation.

## Introduction

Crop protection is an integral part of conserving the yield potential of our crops. Wheat is the dominant crop for human consumption in temperate countries and population growth, coupled with per capita increase in consumption drives demand, which is expected to keep rising (Shewry, 2009; Curtis & Halford, 2014). Currently, aphid damage to crops is controlled mainly by insecticidal treatments (Tanguy & Dedryver, 2009). Insecticide resistance in aphids is now a problem worldwide, with some species being highly resistant to more than one insecticidal class. This, coupled with restrictions on the use of some pesticides in Europe, has focused global research efforts to find alternatives to pesticides, such as the exploitation of natural plant resistance (Reynolds & Borlaug, 2006; Loxdale, 2008; Sparks, 2013).

*Rhopalosiphum padi* (L.), the bird cherry–oat aphid, is a major pest of cultivated cereals, causing yield losses of up to 15% (Leather *et al.*, 1989). Aphids damage plant growth through the removal of nutrients, plant virus transmission (e.g. barley yellow dwarf virus), transmission of toxins via saliva and the reduction of photosynthetic efficiency through the growth of saprophytic fungi on their excreta (Rabbinge *et al.*, 1981). Substantial grain yield losses can result from aphid feeding on early growth stages of winter wheat (*Triticum aestivum* L.); for example, low‐density feeding of *R. padi* can significantly reduce average seed weight (Blackman & Eastop, 1984; Schepers, 1989; Kieckhefer & Gellner, 1992). *R. padi* has a near worldwide distribution, and infests numerous species of *Gramineae* including all major cereal grasses in North temperate regions such as the UK (Blackman & Eastop, 1984). To date, there are no elite hexaploid wheat cultivars that are resistant to *R. padi*, although some rye, *Secale cereale* L., and wheat/rye hybrid Triticale‐derived lines express some resistance (Crespo‐Herrera *et al.*, 2013).

Ancient diploid wheat genotypes have been shown to have the highest resistance to cereal aphid pests, including *R. padi*, *Sitobion avenae* (F.), *Schizaphis graminum* (Rondani) and *Diuraphis noxia* (Kurdjumov) (Deol *et al.*, 1995; Di Pietro *et al.*, 1998; Migui & Lamb, 2003; Elek *et al.*, 2012; Crespo‐Herrera *et al.*, 2013) and provide a useful source of material in the search for resistance mechanisms that ultimately may be utilised in hexaploid breeding programmes. *Triticum monococcum* (L.) lines, in particular, have shown evidence of complete or partial resistance to pathogens and aphids (Spiller & Llewellyn, 1986; Migui & Lamb, 2004; Jing *et al.*, 2007; Elek *et al.*, 2012). Although seldom planted or harvested today, domesticated *T. monococcum* was an agriculturally important crop in the Neolithic through to the Bronze Age (Salamini *et al.*, 2002). The chromosomes of *Triticum* species, such as *T. monococcum,* pair well with the ‘A genome’ of *T. aestivum* potentially allowing introgression of genes from the wild wheats into cultivated species by recombining homologous chromosomes (Valkoun, 2001). It is believed that further genetic improvements to hexaploid wheats may therefore be found by exploring the genetic diversity within *T. monococcum* and discovering new allele variants (Jing *et al.*, 2007).

There are still large gaps in knowledge on the molecular and biochemical mechanisms providing aphid resistance in plants. A more detailed understanding of the genes, regulatory pathways and metabolic components involved are needed (Walling & Thompson, 2012). In many cases, it remains to be elucidated whether resistance is mediated via soluble or structural components of the sieve elements.

Behavioural studies on *T. monococcum* line TM44 indicated a phloem‐based partial resistance to the grain aphid *S. avenae* (Caillaud *et al.*, 1995*a*). Migui & Lamb (2004) hypothesised that the resistance level of *T. monococcum* to *S. avenae* in seedlings could be because of the relative levels of hydroxamic acids (HAs) as reported in Leszczynski *et al.* (1992). Elek *et al.* (2012) observed reduced fecundity by *R. padi* on some *T. monococcum* lines. They found no known HAs in *T. monococcum* lines MDR037 and MDR049, however, but did find unidentified peaks eluting in the same region. Results are conflicting on the role of HAs in plant defences against cereal aphids, with some studies showing a positive correlation (Thackray *et al.*, 1990; Leszczynski *et al.*, 1992; Givovich & Niemeyer, 1994) whereas others find no, or inconclusive relationships between HAs and plant defence (Nicol & Wratten, 1997; Castañeda *et al.*, 2010; Elek *et al.*, 2013).

Recent work in the BBSRC funded LoLa project ‘Enhancing diversity in UK wheat through a public sector pre‐breeding programme’ (BB/I002278/1) identified wheat accessions of *T. monococcum*, exhibiting partial to high resistance to cereal aphids in a high‐throughput screening assay measuring nymph production over 24 h and nymph weight gain over 6 days (manuscript in preparation). This study evaluates in greater depth the variation in the development, fecundity and feeding behaviour of *R. padi* on selected *T. monococcum* lines in comparison with performance on the susceptible hexaploid wheat *T. aestivum* var. Solstice, which was used as a comparative control in the LoLa project. The *T. monococcum* lines selected were MDR049 (showing partial resistance because of the traits affecting nymph weight gain), MDR657 (considered resistant as no nymphs were produced in 24 h) and MDR037 (susceptible). All were tested at the seedling growth stage G11‐12 (Tottman *et al.*, 1979). The electrical penetration graph (EPG) technique (Tjallingii, 2006), where a plant and a wired aphid are connected to an electrical circuit, so that when the aphid stylet penetrates the plant, the circuit is completed and a recording is made which shows the position of the stylet in the plant tissue (Diaz‐Montano *et al.*, 2007), was used to study insect feeding behaviours. In addition, the metabolic phenotypes of the lines were analysed and compared to elucidate the differences in response to aphid feeding damage between susceptible and partially resistant plant lines.

## Materials and methods

### Insect and plant material

A mixed culture of *R. padi*, started from multiple individuals collected from the field at Rothamsted Farm, was reared on barley (*Hordeum vulgare* L. var. Saffron) in ventilated Perspex cages at 20°C, 60–70% relative humidity and 16:8 h light : dark. No specific permission was required for the collection location and no endangered or protected species were involved, all experiments were carried out in controlled laboratory conditions.

*Triticum monococcum* lines MDR037, MDR049 and MDR657 and a commercial variety of *T. aestivum*, Solstice, were sown singly into pots of Rothamsted Prescribed Mix (supplied by Petersfield Products, Leicestershire, UK), which is composed of 75% medium grade (L&P) peat, 12% screened sterilised loam, 3% medium grade vermiculite and 10% grit (5 mm screened, lime free), kept at 22°C, 50% humidity, 16:8 h light : dark regime and watered daily. Seven‐day‐old seedlings of growth stage GS11‐12 (Tottman *et al.*, 1979) were used for all the experiments. All plant material used was grown in the same conditions.

### Aphid development and fecundity assays

Development and fecundity assays were conducted on the three *T. monococcum* lines and *T. aestivum* var. Solstice under controlled conditions (22°C, 16:8 h light : dark). Replicate batches of four alate *R. padi* were collected from the roof of the rearing cage and put into 2 cm diameter clip cages, as described by MacGillivray and Anderson (1957), one of which was then attached to the first true leaf of each of the 10 replicate plants of a *T. monococcum* line or of Solstice in a randomised design. The alates were removed after 24 h and the number of nymphs produced was recorded. Nymphs were allowed to develop on the plants for 7 days, after which the survivors were transferred to 0.2 mL Eppendorf tubes and weighed on a microbalance (Cahn 33; Scientific and Medical Products Ltd, Manchester, UK) and the mean nymph weight was calculated. An individual nymph was selected at random from each batch and returned, in the clip cage, to the plant. The nymphs were monitored at the same time each day until they produced their first nymph (time *D*). Subsequent nymphs were removed and recorded daily over a period equivalent to time *D*. From the data, the intrinsic rate of population increase (rm) was calculated using the formula devised by Wyatt and White (1977), that is

$$rm=C\left(\ln\left(FD\right)/D\right)$$

where the constant *C* = 0.74 is a correction factor, *D* is the preproductive period (days) and FD is the number of nymphs produced in the reproductive period equal to *D*. Data were analysed using a linear mixed model fitted by restricted maximum likelihood (REML) in GenStat (16th edition; VSN International, Hemel Hempstead, UK).

### EPG recordings

Feeding behaviour of *R. padi* was studied by EPG using the methodology of Tjallingii (1988, 2000). Apterous adults were collected on the day of the experiment, starved for approximately 1.5 h and then attached to a 25‐µm piece of 1.5–2 cm long gold wire with the aid of a specially adapted suction pump and water‐based adhesive containing silver paint. The paint was also used to connect the gold wire to a piece of 2.5–3 cm copper wire, which was connected in turn to a brass pin via solder. This apparatus was then connected to an 8‐channel ‘Giga‐8’ DC amplifier of 1 GΩ input resistance (EPG‐systems, Wageningen, the Netherlands) housed in a grounded Faraday cage. The first leaf of a 7‐day‐old wheat plant was secured to the base of an upside down 100‐mL glass beaker using two pieces of clear plastic tape (2.5 cm × 0.5 cm) on the two edges where the leaf blade met the circumference to restrict plant movements without applying pressure to the leaf blade itself. Each stage of this procedure was repeated for the eight replicates.

A Petri dish filled with water was placed under each pot and the plant was watered so that the soil was saturated to ensure good electrical conductivity throughout the duration of the experiment. A plant electrode was then placed in the soil, the aphid put on the plant and an 8‐h EPG recording was commenced using Stylet+ data acquisition software (EPG‐systems). All recordings were made between 11:00 and 20:00 h, with room temperature maintained at 21 ± 1°C and a constant light level provided by three 80‐W fluorescent lights. Two replicates of each of the four lines were run per day and the positions of the plants and probe wires were randomised. A total of 136 replicates were tested.

### EPG data processing and analysis

The EPG waveform recordings were interpreted using the Stylet+ analysis software, annotated and imported into version 10.6m of the EPG analysis Microsoft Excel macro (available from Dr Schliephake via EPG‐systems) to calculate feeding behaviour parameters from the waveforms.

Aphid waveforms were placed into the following categories: non‐probing (Np), stylet pathway phase containing waveforms A, B and C (C), phloem sieve element salivation (E1), phloem sieve element ingestion (E2), derailed stylet mechanic/penetration difficulties (F) and xylem drinking (G). Extracellular phloem salivation (E1e) was not found in this study (Tjallingii, 1988, 2000; Pettersson *et al.*, 2007).

Each response was analysed separately, using a linear mixed model fitted using REML in GenStat (16th edition; VSN International, 2013). Where appropriate, the response variables were transformed to common logarithms to conform to the assumptions of the analysis (variance homogeneity and normality of residuals). The variables with zeros required an offset to be added before taking logs; these were set at half the minimum non‐zero value recorded. Hypothesis testing was carried out at the 5% significance level. Responses were considered valid if feeding activity was recorded within the first hour and feeding activity was recorded for 30 min or longer within the last hour of recording. Invalid responses were excluded from analysis leading to unequal replication of varieties (MDR037 *n* = 16, MDR049 *n* = 25, MDR657 *n* = 24 and Solstice *n* = 23).

### Metabolomics sample preparation

For aphid‐treated plants, 20 alate aphids were put onto the first true leaf of each plant at 10:00 h, inside a 2‐cm diameter clip cage and left to feed for 24 h before sampling. Control plants were treated identically, except they had an empty clip cage placed on the first true leaf. Three replicate plants of each treatment were randomised and grown singly in each pot. Prior to sampling, aphids were removed from the leaf. Plant tissue was sampled from the area where aphids were feeding (localised response, within the clip cage) as well as up‐ and down‐stream from the feeding site (systemic response, proximal and distal from the clip cage). Systemic tissue was collected from a different set of treated plants from localised tissue, but pooled so that plant tissue proximal and distal from the clip cage for each plant was put in the same vial. Set‐up and sampling was carried out within a 30‐min period. Plant material for metabolomics was snap‐frozen in liquid nitrogen to quench metabolism.

Preweighed aliquots (3.0 ± 0.5 mg) of each freeze‐dried tissue sample were milled (5 min) in 2‐mL round bottom Eppendorf tubes with one tungsten carbide bead, using Retsch MM300 Mixer Mill MM 300 (Retsch, Haan, Germany). Deuterium oxide–deuterated methanol (D_2_O‐CD_3_OD) (4:1 v/v) incorporating 0.01% w/v d_4_‐trimethylsilyl propanoic acid (TSP) (1.0 mL) was added to each tube. After mixing, the tubes were heated to 50°C for 10 min, cooled and centrifuged. Supernatant (850 µL) was transferred from each tube to a clean Eppendorf tube and then heated to 90°C for 2 min. The samples were then cooled to 4°C for 30 min and centrifuged. The supernatant (700 µL) was removed to a clean Eppendorf tube and mixed with 20 µL of deuterated 2.6 M potassium phosphate buffer, pH 7.4 and 10 µL of 32 mM ethylenediaminetetraacetic acid solution in D_2_O. This buffered sample (650 µL) was transferred to a 5‐mm nuclear magnetic resonance (NMR) tube for analysis.

### 1D ^1^H NMR data collection and data analysis

The 1D ^1^H NMR spectral acquisition was carried out on an Avance 600 MHz NMR Spectrometer (Bruker Biospin, Coventry, UK) using parameters and settings as described previously (Ward *et al.*, 2010). Briefly, 1D ^1^H NMR spectra were acquired at 300 K using a 5‐mm selective inverse (SEI) probe. A water suppression pulse sequence (noesygppr1d) was used, employing a 90° excitation pulse angle and a presaturation pulse during the relaxation delay of 5 s. Data were acquired using 128 scans of 65 536 data points across a sweep width of 12 ppm. The 1D ^1^H NMR free induction decays (FID) were zero filled to double their original size, and Fourier transformed with an exponential window function (0.5 Hz). Spectra were manually phased and automatically baseline corrected in Amix (Analysis of MIXtures; Bruker Biospin) using a second‐order polynomial. ^1^H chemical shifts were referenced to d_4_‐TSP at δ0.00 and spectra were automatically reduced to create an ASCII file containing integrated regions of equal width (0.01 ppm). Spectral intensities were scaled to the d_4_‐TSP region (δ0.05 to −0.05). The ASCII file was imported into Excel for the addition of sampling/treatment details. The regions for unsuppressed water (δ4.865–4.775), d_4_‐MeOH (δ3.335–3.285) and d_4_‐TSP (δ0.05 to 0.05) were removed prior to importing the data set into SIMCA‐P 13.0 (Umetrics, Umea, Sweden) for multivariate analysis. For statistical analyses, technical replicates were averaged and standard deviations were displayed on the basis of three biological replicates. Annotation of peaks to individual metabolites was achieved via comparison with a library of authentic standards prepared in identical conditions to the test samples and run under identical 1D ^1^H NMR conditions. Characteristic regions for each metabolite were used for quantification against a known concentration of d_4_‐TSP, which was added to every sample. Concentrations were expressed on an mg g^−1^ dry weight (d.w.) basis using known starting weights of each tissue sample.

The 1D ^1^H NMR data was collected on wheat leaf tissues according to procedures described by Ward *et al.* (2010) and Corol *et al.* (2014). Known metabolites present in the ^1^H NMR spectra were quantified against an internal standard and compared using multivariate analysis. Principal component analysis (PCA) was carried out in SIMCA‐P 13.0 (Umetrics) using the quantified (mg g^−1^ d.w.) data matrix using unit variance scaling. The PCA score plots were generated to visualise groupings in the samples and contribution plots were made to explain discriminatory metabolites between groupings.

## Results

### Aphid development and fecundity

**Figure 1:**
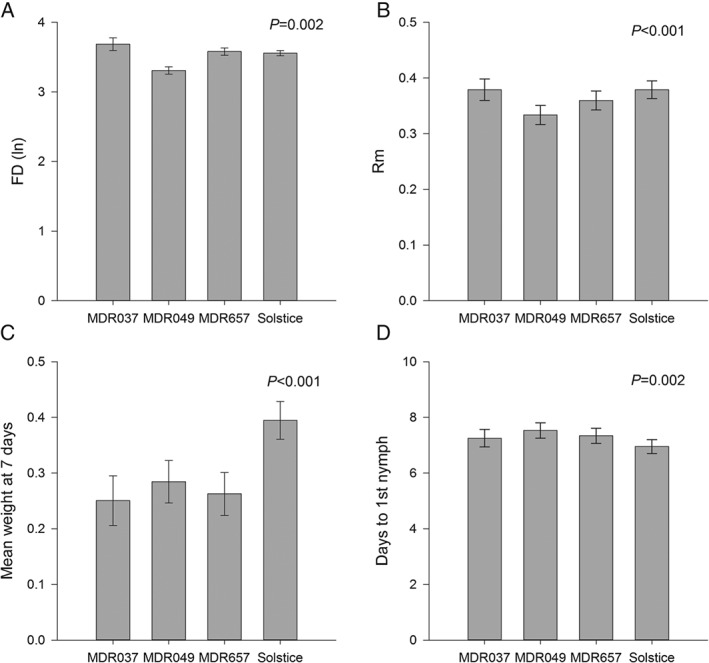
(A) FD: number of nymphs produced per adult aphid over a period equivalent to that of its developmental time (B) Rm: intrinsic rate of increase (C) mean weight (mg) at 7 days and (D) number of days to first nymph. Data were analysed using a linear mixed model fitted by restricted maximum likelihood. Error bars represent ± SEM.

The number of nymphs produced per adult aphid over a period equivalent to that of its developmental time (*D*) is represented as FD. In the experiments described in this study, FD differed according to the wheat cultivar, with aphids on MDR049 producing fewer nymphs on average than the other varieties (Fig. 1A). The average intrinsic rate of population increase (rm) and the average number of days until the first nymph was produced (*D*) also differed among the varieties, with MDR049 having the lowest rate of increase and the longest time to first nymph (Fig. 1B and Fig. 1D). Nymph weight also differed, with the diploid *T. monococcum* (MDR) varieties having lower aphid weights at 7 days than the hexaploid Solstice, where many nymphs had already moulted to adults (Fig. 1C).

### Electrical penetration graph

**Figure 2:**
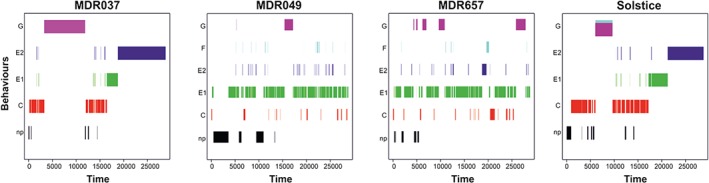
Continuous time (s) recordings for a representative replicate of each of four varieties. Behaviours: np: not probing (black), C: pathway phase (red), E1: salivation (green), E2 phloem ingestion (dark blue), F: derailed stylet mechanics (light blue), G: xylem ingestion (purple).

Overall, the main differences were between MDR037 and Solstice versus MDR657 and MDR049, which became apparent on analysis of the different feeding phases (Fig. 2). Statistical results are all summarised in Table 1. The smaller number of replicates of MDR037 was because of the aphids not feeding for 30 min or longer during the last hour of recording.

**Table 1:** List of electrical penetration graph variables. Total duration (in seconds), frequency and average duration (predicted means) from 8 h of recording of Rhopalosiphum padi feeding on Triticum monococcum lines MDR037, MDR049, MDR657 and Triticum aestivum var. Solstice

Variables	MDR037	MDR049	MDR657	Solstice	F pr	Transformation
Sample size of qualifying replicates	16	25	24	23		
Age of replicates qualifying (%)	47.06	73.53	70.59	67.65		
*Probing (tissue penetration)*						
Number of probes	2.764	3.881	3.573	3.353	<0.001	Sqrt
Number of brief probes (<180 s)	1.021	1.958	1.48	1.567	0.025	Sqrt
Average probe length	3.548	3.226	3.304	3.391	<0.001	Log
Total time probing	25 691	24 071	24 868	25 117	0.245	None
Time to first probe	2.398	2.275	2.399	2.457	0.285	Log
Duration of first probe	49.81	36.02	54.9	38.48	0.066	Sqrt
*Pathway*						
Number of pathway periods (C)	20.99	33.09	32.64	24.39	<0.001	None
Average time in pathway (C)	462.8	450.2	487.5	450.8	0.746	None
Time to first potential drop (pd) (from start of first probe)	2.176	1.866	1.786	2.136	0.434	Log
Potential drops (pds) to the first phloem event (E)	1.188	1.398	1.318	1.39	0.037	Log
*Xylem drinking*						
Number of xylem drinking (G)	0.963	1.297	1.16	0.729	0.023	Sqrt
Average xylem drinking (G)	43.6	39.68	39.22	49.3	0.285	Sqrt
Time to first xylem drinking (G)	113.1	108.7	106.6	132.3	0.176	Sqrt
*Salivation and phloem feeding*						
Number of single salivation event (sgE1)	1.986	2.596	2.814	1.771	0.001	Sqrt
Average single salivation event (sgE1)	1.682	1.689	1.73	1.99	<0.001	Log
Number of salivation events (E1)	3.713	4.014	4.392	3.217	0.013	Sqrt
Average salivation event (E1)	1.665	1.678	1.679	2.032	<0.001	Log
Time to first salivation (E1)	60.5	73.38	72.01	78.04	0.3	Sqrt
Number of phloem feeding events (E2)	2.457	2.214	2.502	2.075	0.316	Sqrt
Average phloem feeding event (E2)	2.96	2.021	2.247	2.775	<0.001	Log
Total phloem feeding event (E2)	3.696	2.65	3.011	3.355	<0.001	Log
Maximum phloem feeding event (E2)	3.567	2.403	2.776	3.177	<0.001	Log
Number of sustained phloem events (sE2)	1.2471	0.3607	0.3758	0.7842	<0.001	None
Time to first phloem feeding (E2)	72.17	98.15	82.13	90.39	0.107	Sqrt
Time to first phloem feeding from 1st salivation	2.271	2.831	2.47	2.531	0.395	Log
Time to first sustained phloem feeding (sE2)	16 981	26 514	24 235	20 724	<0.001	None
Number of probes to first phloem feeding (E2)	1.116	1.885	1.34	1.715	0.029	Sqrt
Number of probes to first sustained phloem feeding (sE2)	2.153	3.155	2.62	2.693	0.19	Sqrt

### Probing (tissue penetration)

No difference was found in the average length of time it took *R. padi* to probe the lines tested for the first time, or in the average duration of the first probe. However, the average number of probes by the aphids differed between the lines, with fewer probes on MDR037 than on MDR049 and MDR657, and a higher number of brief probes (lasting less than 3 min) on MDR049 than on MDR037. There was a difference in average probe time, where again the aphids spent longer in the plant tissue of MDR037 than in MDR049 and MDR657. However, the total length of time the aphids spent within the plant tissue during the 8‐h recording did not vary among the lines (Table 1; Fig. 2).

### Pathway phase and reaching the phloem

The number of pathway periods was higher on MDR049 and MDR657 than on MDR037 and Solstice, but there was no difference in the average length of time spent in pathway phase. A higher number of potential drops (stylet entry into a non‐target cell) were observed in MDR049 and Solstice compared with MDR037, but no difference was found in the time to the first potential drop within a probe with a potential drop.

The number of brief probes lasting less than 3 min before the first phloem event and the time it took the aphids to initiate the first phloem ingestion did not differ among the lines.

### Salivation and phloem ingestion

A difference was observed in the number of times aphids salivated without ingesting phloem contents (single salivation events), which were higher on MDR049 and MDR657 than on Solstice. However, the average salivation time was higher on Solstice than on the MDR lines. When considering all salivation events, *R. padi* salivated fewer times on Solstice than on MDR657, but no difference was observed between the other lines. Again, the average time spent salivating was higher for Solstice than for the MDR lines, but there was no difference in the time to first salivation by the aphids.

No difference was observed neither in the number of times the aphids engaged in phloem ingestion, nor in the time to first phloem feeding by the aphids on the different lines. However, *R. padi* probed more often on MDR049 before the first phloem feed than on MDR037 and spent longer on average phloem feeding on Solstice and MDR037 than on MDR049 and MDR657. There was also a difference in total time spent phloem feeding, where *R. padi* spent longer on MDR037 than on MDR049 and MDR657. A difference was also present between MDR049 and Solstice. The longest time spent phloem feeding was on MDR037, which differed from MDR049 and MDR657. The aphids also had a longer feeding period on Solstice than on MDR049. There was no statistically significant difference in the time from first phloem salivation to first phloem feeding between the lines.

*Rhopalosiphum padi* had more sustained phloem feeding periods of over 10 min on MDR037 than on MDR049 and MDR657. No significant difference was found in the number of probes to the first sustained phloem feeding (E2), but time to the first sustained phloem feeding was found to differ, where *R. padi* started sustained phloem feeding more quickly on MDR037 than on MDR049 and MDR657. It also happened faster on Solstice than on MDR049.

### Xylem phase

There was a significant difference in the number of times *R. padi* engaged in xylem drinking, which was higher on MDR049 than on Solstice. However, the period to the first time the aphids drank from the xylem and the average time spent xylem drinking did not differ between lines.

### Metabolomics

**Figure 3:**
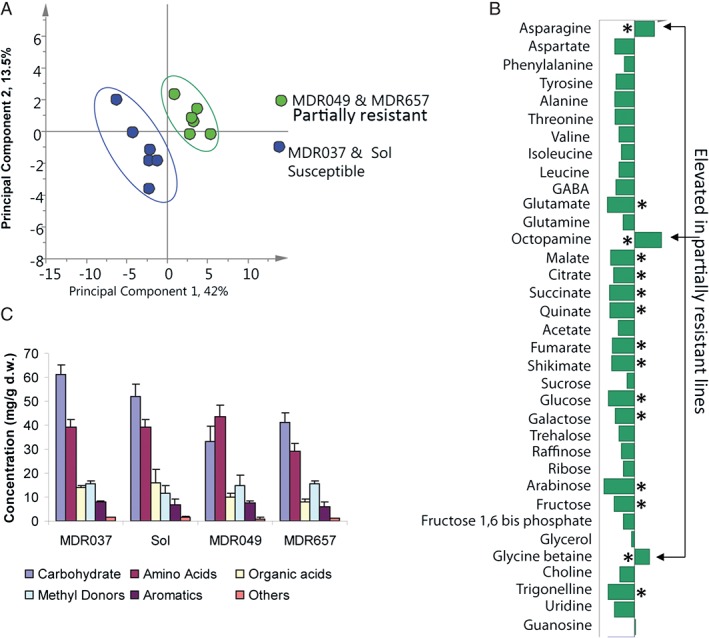
Metabolomics comparison of 1D ^1^H nuclear magnetic resonance data from wheat leaf tissue. (A) Principal component analysis scores plot showing separation between partially resistant (green) and susceptible (blue) lines. (B) Contribution plot describing metabolite differences in partially resistant plants MDR049 and MDR657. (C) Comparison of total metabolite concentrations by chemical class for each line. *P < 0.05

The PCA of the four lines showed a separation in PC1 (42%) into two distinct clusters with MDR049 and MDR657 in one cluster and MDR037 and Solstice in the other (Fig. 3A). Analysis of the contribution plot (Fig. 3B) showed that the partially resistant lines MDR049 and MDR657 contained lower levels of the majority of primary metabolites including those in the classes carbohydrates, organic acids, amino acids and aromatics. Exceptions were asparagine, glycine betaine and octopamine, which were all increased in MDR049 and MDR657 compared with Solstice and MDR037. Summation of the concentration of each known metabolite into compound classes (Fig. 3C) showed that carbohydrates and amino acids dominated the wheat leaf metabolome. Total carbohydrates were lower in MDR049 (33 ± 6 mg g^−1^ d.w.) and MDR657 (41 ± 4 mg g^−1^ d.w.) compared with the susceptible lines (61 ± 4 and 52 ± 5 mg g^−1^ d.w.). In MDR049, the levels of total amino acids were higher (44 ± 5 mg g^−1^ d.w.) than soluble carbohydrate, although this was not the case for MDR657. Total organic acids were reduced in MDR049 (10 ± 2 mg g^−1^ d.w.) and MDR657 (8 ± 1 mg g^−1^ d.w.) compared with Solstice (16 ± 6 mg g^−1^ d.w.) and MDR037 (14 ± 0.5 mg g^−1^ d.w.) (Table S1, Supporting information).

**Figure 4:**
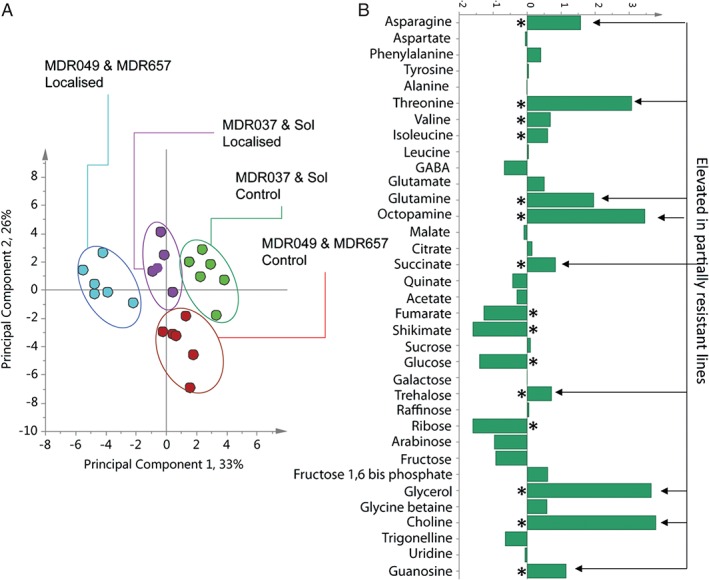
Metabolomics comparison of 1D ^1^H NMR data from wheat leaf tissue showing separations between control and aphid‐localised tissue. (A) Principal component analysis scores plot showing separation between partially resistant–control (red), susceptible–control (green), susceptible–aphid‐localised (purple) and partially resistant–aphid‐localised (turquoise) lines. (B) Contribution plot describing metabolite differences between resistant lines MDR049 and MDR657 after aphid infestation. *P < 0.05

Further experiments measured the changes in the metabolome after aphid feeding. The PCA of the ^1^H NMR data (Fig. 4) showed that leaf samples, fed on by aphids, separated from the control group (Fig. 4A), indicating a generalised response to aphid infestation. The greatest separation, however, was seen in lines MDR049 and MDR657 following the introduction of aphids. These showed a distinct cluster in the direction of PC1 (33%) and these samples were separated from their controls and the aphid‐treated tissue from susceptible lines. The contribution plot (Fig. 4B; Table S1) describing the metabolite differences responsible for this separation indicated a number of metabolites, across different compound classes that were elevated in tissue from partially resistant lines following the aphid localisation. Significant differences included an increase in the amino acids asparagine, glutamine, octopamine and threonine. Reductions in fumarate and shikimate were accompanied by an increase in succinate following aphid localisation. No differences in sucrose, the major soluble carbohydrate present in young wheat leaves, were observed. But there was an increase in trehalose following aphid localisation on the partially resistant lines, accompanied by a reduction in glucose, fructose and ribose. Of the other metabolites that could be quantified in this study, large increases in glycerol and choline were evident in lines MDR049 and MDR657 following aphid localisation (Table S1).

**Figure 5:**
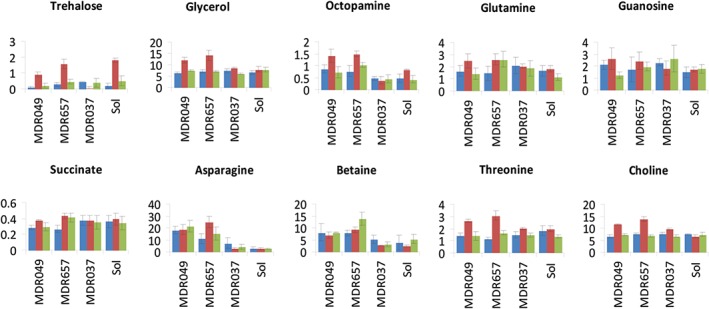
Histograms showing metabolite concentrations (mg g^−1^ d.w.) in wheat leaf tissue, derived from 1D ^1^H NMR. Error bars show SD. Blue, control; red, aphid‐localised; green, systemic response. Note different scales on y‐axis.

Inspection of the absolute concentration data for each line under control, systemic or aphid localisation events allowed the generalised aphid response to be discerned from the additional response seen in the partially resistant lines. In terms of individual carbohydrates (Fig. S1), sucrose levels increased in all lines following the introduction of aphids to the leaves. In contrast, glucose and fructose decreased in response to aphids. For the less abundant carbohydrates, no change was seen in arabinose or galactose, but raffinose and trehalose showed significant increases in all lines, except MDR037, upon the introduction of aphids (Figs 5 and S1). The sugar alcohol, glycerol, was present at low concentration (approximately 6 mg g^−1^ d.w.) in all lines and there was no difference in concentration in lines MDR037 and Solstice in response to aphids. Levels of glycerol did, however, rise in MDR049 and MDR657 to approximately 14 mg g^−1^ d.w. in response to aphid localisation (Fig. 5; Table S1). This increase was only seen at the point of aphid localisation and was not observed in the systemic tissues.

Levels of the major organic acids, citrate, malate, quinate and fumarate were lower in the partially resistant lines MDR049 and MDR657 compared with Solstice and MDR037 (Fig. S1; Table S1). The largest difference was observed in fumarate where levels in susceptible control tissues were 2 and 4 mg g^−1^ d.w. in MDR037 and Solstice, respectively. In the partially resistant lines, concentrations of fumarate were just 0.3 mg g^−1^ d.w. In the less abundant organic acids, small increases were found in succinate in both MDR049 and MDR657 in response to aphids (Fig. 5; Table S1). Acetate was at comparable levels in all control plants and decreased in the aphid‐localised tissue. Similarly, shikimate decreased in the aphid‐localised tissue, most evident in lines MDR049 and MDR657 although a similar, but less pronounced, reduction was seen in MDR037 (Fig. S1; Table S1).

The levels of the majority of amino acids did not discriminate between the four lines in this study (Figs 5 and S2). Exceptions were gamma‐amino butyric acid (GABA), which although at similar levels (3–4 mg g^−1^ d.w.) in all control plants, showed a slight decrease in all lines in response to aphid localisation (Fig. S2). Levels of threonine were also similar in all control plants at around 1 mg g^−1^ d.w. and no significant changes were observed after the introduction of aphids to lines Solstice and MDR037. However, in the partially resistant lines MDR049 and MDR657, threonine levels rose to approximately 2.5 mg g^−1^ d.w. when aphids were applied to the plants (Fig. 5). Asparagine levels were low (2–3 mg g^−1^ d.w.) in susceptible lines, in contrast to the levels observed in the partially resistant lines where asparagine levels were markedly higher even in control tissue (18 mg g^−1^ d.w. in MDR049) and which did not decrease following introduction of aphids to the tissue. Of the other metabolites which discriminated between the four lines, glycine betaine was higher in lines MDR049 and MDR657 but did not increase further on the introduction of aphids to the leaf tissue. In contrast, choline was present at similar levels in all four control samples (approximately 5 mg g^−1^ d.w.) but doubled on the introduction of aphids to lines MDR049 and MDR657 and octopamine was detected in all lines but was elevated in the partially resistant lines. This metabolite was further elevated after aphid localisation in lines MDR049 and MDR657 (Fig. 5).

## Discussion

The results from our development assays confirmed previously observed reduced susceptibility of *T. monococcum* MDR049 to *R. padi*, but ‘resistance’ of MDR657 did not last beyond the 7‐day seedling stage. Development assays differ from the other methods used here in that they span over approximately 2‐week duration during which the plant chemistry is likely to vary with plant development. Feeding behaviour on 7‐day‐old seedlings partially supported the above‐mentioned fact that *R. padi* probed more often on MDR049 before the first phloem feed and spent longer phloem feeding on Solstice and MDR037 than on MDR049 and MDR657, indicating probability of reduced development at this growth stage. Metabolomic PCA on tissue from 7‐ to 8‐day‐old seedlings showed that the partially resistant lines contained lower levels of the majority of primary metabolites including those in the classes carbohydrates, organic acids, amino acids and aromatics.

In line with work carried out previously by Elek *et al.* (2012), the bioassays reported here confirmed the reduction in fecundity and development of the aphid *R. padi* feeding on the *T. monococcum* line MDR049, and our observation of partial resistance owing to traits affecting aphid fitness, in the initial screen. In contrast, the apparent resistance to *R. padi*, observed in our initial screen of line MDR657, was found to be a transient effect only, highlighting the need for detailed study of lines selected by high throughput screening. There was no reduction in development and fecundity on MDR657 compared with the control, Solstice. In the initial screen, replicate batches of two alate *R. padi* produced no nymphs on MDR657 over 24 h (unpublished), but in these development assays replicate batches of four alates did produce a few nymphs, which went on to develop relatively normally. This indicates that 7‐day‐old seedlings of MDR657 have deterrent effects against aphids, but this effect is not sustained as the plants age (the plants were approximately 21 days old by the end of the assay). Elek *et al.* (2013) found that young 6‐day‐old seedlings of hexaploid wheat lines tested, had the greatest levels of chemical defences (HAs), which declined as the plants aged to 13 days. The same authors (Elek *et al.*, 2012) found no known HAs in the *T. monococcum* lines they tested, but this does not rule out a decline in plant defence with age, which could account for the result with MDR657. MDR049 seedlings were less effective at deterring nymph production over 24 h, but apparently retained some defence over a longer period, resulting in a reduction in population increase. The PCA of the comparative metabolomics profiles of MDR657 and MDR049 at 7 days showed clear differences to the susceptible lines. A comparison of the chemical changes in the two lines over time may help to clarify the differences between them since characteristics that both deter early nymph production and reduce aphid fitness would be useful in reducing the susceptibility of wheat to aphid attack.

It was also shown that the aphids were heavier on the hexaploid, Solstice, than on the *T. monococcum* lines. This difference was exacerbated as, because of faster development, many of the nymphs on Solstice had already moulted to adult by day 7 and therefore this could also indicate the different suitability of these hexaploid versus diploid plants for aphid development.

The results from the EPG indicate that the partial resistance observed in the *T. monococcum* lines MDR049 and MDR657 is localised in the phloem and this is in line with other evidence from numerous studies showing that most of the primary mechanisms for host‐plant resistance occur in the phloem (Walling & Thompson, 2012). In this work, it was demonstrated by an increase in the number of probes before the first phloem feed, a higher number and duration of salivation events without subsequent phloem ingestion and a shorter time period spent feeding on the partially resistant plants. An increase in salivation events, without subsequent phloem ingestion, could indicate transient difficulties for the aphid in switching between the two behaviours to begin phloem ingestion, perhaps because of phloem occlusion, or a toxic effect preventing the aphids from feeding for sustained periods (Tjallingii, 2006; Dinant *et al.*, 2010). No difference was found neither in the time taken to the first probe, nor in the total time spent probing (i.e. within plant tissues), further suggesting that the partial resistance is not antixenotic, but more likely of a trait affecting aphid fitness at this growth stage. Neither did we see a difference between the lines in length of time to initiate phloem feeding on partially resistant *T. monococcum* lines. These results are in line with other work on *T. monococcum* and *T. aestivum* where the time taken to initiate probing, or first phloem contact, did not differ (Caillaud *et al.*, 1995*b*; Spiller, 1988). Also of note is that the time to reach the first salivation (E1) or phloem ingestion (E2) phases did not differ between the varieties in this study, but it took the aphids longer to reach the first sustained phloem ingestion phase on MDR049. This is further evidence that the aphids have problems engaging in an ingestion phase after salivation and that when this is achieved they find it harder to maintain ingestion in these varieties.

During probing, an aphid secretes two types of saliva, one is proteinaceous and forms the stylet sheath to aid the stylet movement in the plant tissues, whilst the other is a watery saliva. It is thought that salivation of watery saliva into the sieve elements during feeding suppresses phloem wound responses, preventing blockage of the sieve elements (Pettersson *et al.*, 2007). Some research effort has gone into studying defence responses to aphids by plants and a number of mechanisms have been suggested, such as forisomes, elongate proteins bodies, which are thought to disperse upon wounding and callose formation acting as occlusion mechanisms, or the production of secondary metabolites such as HAs (Will *et al.*, 2013). We did not detect HAs in our samples, but this could be because levels were below detection threshold. Results on whether the levels of defensive compounds, for example phenolics, are important to aphid defences are contradictory (Akbar *et al.*, 2014). In this study, the only indication for a physical or chemical barrier detected before phloem contact with sieve elements was a higher number of short probes of less than 3 min on MDR049 compared with the other lines. Garzo *et al.* (2002) reported similar behaviour in *Aphis gossypii* feeding on resistant melon (*Cucumis melo*) genotypes.

Clear metabolic phenotypes separate the partially resistant and susceptible wheat lines in our study, with a number of compounds being detected at different levels in the susceptible and partially resistant lines, and others showing an accumulation following aphid infestation. Both susceptible and partially resistant plants showed a response to aphid infestation in localised tissue, but there was no clear systemic response. The function of compounds which accumulate or are reduced is of interest in understanding how the changes may relate to the interaction taking place between the aphid and the plant, although we point out that our results are based on whole‐leaf samples and total leaf concentrations may not be a direct reflection of those found in phloem sap.

Nutritional quality is a major factor in the survival and development of aphids. Lower levels of primary metabolites such as carbohydrates would make MDR049 and MDR657 less suitable as hosts. Phloem‐mobile sugars are the principal source of carbon for aphid development and aphids are known to show a compensatory feeding response, ingesting food at an inverse rate to the concentration of sucrose (Douglas *et al.*, 2006; Douglas & van Emden, 2007), so the reduction in time the aphids spend phloem feeding on these lines is contradictory and could suggest an occlusion mechanism, or a toxic or antifeedant effect. However, as stated above, the total leaf sugar concentrations measured in the metabolic study are not a direct reflection of phloem sugars.

Compounds which are at a higher level in partially resistant lines and/or induced on aphid infestation are also of interest, as they might hold the clue to the cause of the resistance. Not all of the compounds identified in this study have previously been associated with aphid resistance, although some are associated with pathogen attack. This is not surprising, as the feeding behaviour of aphids via a stylet, which they thread through the plant to find the sieve elements, is in many ways similar to pathogen attack as seen in changes in RNA profiles and volatile emissions (Kaloshian & Walling, 2005).

Glycerol was found in higher levels in the localised plant tissue samples from our aphid infested plants, of partially resistant *T. monococcum* lines MDR049 and MDR657, than from systemic tissue or control plants. Glycerol has been shown previously to induce systemic acquired resistance in the cacao tree, *Theobroma cacao*, to the cacao pathogen *Phytophthora capsici* via foliar application, which induced the endogenous levels of glycerol‐3‐phosphate (G3P) (Zhang *et al.*, 2014). It can be converted into G3P by the enzyme glycerol kinase, which in turn is a well‐known inducer of a broad‐spectrum immunity in plants (Chanda *et al.*, 2008). The effect of induced glycerol in localised tissue of aphid infested partially resistant *T. monococcum* plants needs to be elucidated.

Trehalose was also found to be elevated in partially resistant lines MDR049 and MDR657 as well as the hexaploid Solstice on aphid infestation. Trehalose is a disaccharide, which is found in the majority of plants in only trace amounts and has been thought to control basic plant processes as well as being involved in resistance to abiotic stress factors and signalling. More recent work is now showing that it is trehalose‐6‐phosphate, a precursor of trehalose, which is the signalling molecule (Paul *et al.*, 2008; O'Hara *et al.*, 2013) and interestingly it has been shown that treating wheat with trehalose can induce partial protection against the powdery mildew pathogen (*Blumeria graminis*), which could be because of the activation of defence responses (Reignault *et al.*, 2001; Tayeh *et al.*, 2014). Trehalose is important to insects, as the starting point in chitin synthesis (Paul *et al.*, 2008) and is known to occur in the haemolymph of the aphids *Megoura viciae* and *Acyrthosiphon pisum* (Rhodes *et al.*, 1997). Feeding by *Myzus persicae* has been shown to induce accumulation of trehalose and this is thought to be critical in defence responses in *Arabidopsis thaliana* to aphids (Hodge *et al.*, 2013). The saliva of *D. noxia*, *A. pisum* and *Phlebotomus arabicus* has been found to contain trehalase, which converts trehalose to two glucose molecules and it has been suggested that the role of trehalase in the saliva of the aphids is to disrupt the stress response signalling by the plant (Nicholson *et al.*, 2012; Hodge *et al.*, 2013). Another suggested role could be that the aphids are able to interfere with the sugar sensing pathway of the plant, manipulating it in their favour as has been suggested with plant pathogens (O'Hara *et al.*, 2013). Thus, our finding of elevated trehalose in resistant wheat warrants further investigation.

Asparagine is a key amino acid for the storage and transport of nitrogen in plants and accumulates under biotic and abiotic stress conditions (Lea *et al.*, 2007). In this study, levels of asparagine were higher in the partially resistant line MDR049, but were not elevated further on aphid infestation. Asparagine has been shown to be induced on pathogen infection such as that of *Pseudomonas syringae*‐infected tomato leaves. There it was coupled with increased asparagine synthetase activity; this catalyses the conversion of glutamine into asparagine, suggesting that asparagine is exported from plant cells in response to infection or when senescence or N deficiency occurs (Olea *et al.*, 2004; Rico et al., 2009). Kazemi & van Emden (1992) also observed reduced fecundity of *R. padi* on *T. monococcum* and found a correlation with leaf concentrations of three amino acids, where increase in threonine was associated with reduced aphid fecundity. This concurs with our results, which showed that threonine was also elevated in aphid infested localised plant tissue.

Octopamine functions as a neuromodulator, neurotransmitter and neurohormone in insect nervous systems, prompting the organism to dynamic action and initiating a variety of effects such as agonistic effects on the heart rate of the honeybee, *Apis mellifera macedonica*, and the olive fruit fly, *Bactrocera oleae* (Papaefthimiou & Theophilidis, 2011). It has also been shown to increase in concentration in locusts in response to stress (Verlinden *et al.*, 2010). The role of induced octopamine in response to feeding by *R. padi* in our study is unclear, but interestingly it has also been shown to accumulate during the interaction of tomato plants with the bacterial pathogen *P. syringae* pv. *tomato* (López‐Gresa *et al.*, 2011), so there could be a role in stress response.

We can conclude that, based on the results reported here and what is available in the scientific literature, which ranges over different plant and aphid species, the evidence is building for a phloem‐localised resistance in plants to aphids. What is interesting is that the compounds that are present at higher levels and/or accumulate in partially resistant plants with or without feeding by *R. padi* are compounds that also increase in response to pathogens. The feeding effect of these compounds now needs to be studied to elucidate any negative effect on aphid survival or positive effects on plant resistance, tolerance and interaction with viruses vectored by these stealthy herbivores. The aim of this work is to improve crop protection, hence elucidating the mechanisms for aphid resistance in wheat to be used in breeding programmes and/or identifying compounds, which could be used to control aphids, could ultimately contribute to a more sustainable agriculture.

## Supporting information


**Figure S1.** Histograms showing metabolite concentrations (mg g^−1^ d.w.) of carbohydrate and organic acids in wheat leaf tissue, derived from 1D ^1^H nuclear magnetic resonance. Error bars show SD. Blue, control; red, aphid‐localised; green, systemic response. Note different scales on y‐axis.

**Figure S2.** Histograms showing concentrations (mg g^‐1^ d.w.) of amino acid, methyl donor and aromatic metabolites in wheat leaf tissue, derived from 1D ^1^H nuclear magnetic resonance. Error bars show SD. Blue, control; red, aphid‐localised; green, systemic response. Note different scales on y‐axis.

**Table S1.** Results from analysis of variance for metabolomic phenotyping of Triticum monococcum lines MDR049, MDR657, MDR037 and Triticum aestivum var. Solstice
