# Supplementary figures and images for: Triticum monococcum lines with distinct metabolic phenotypes and phloem‐based partial resistance to the bird cherry–oat aphid Rhopalosiphum padi
Source: Ann Appl Biol. 2016 Feb 29;168(3):435–49. doi: 10.1111/aab.12274 (PMC4982108; doi:10.1111/aab.12274)

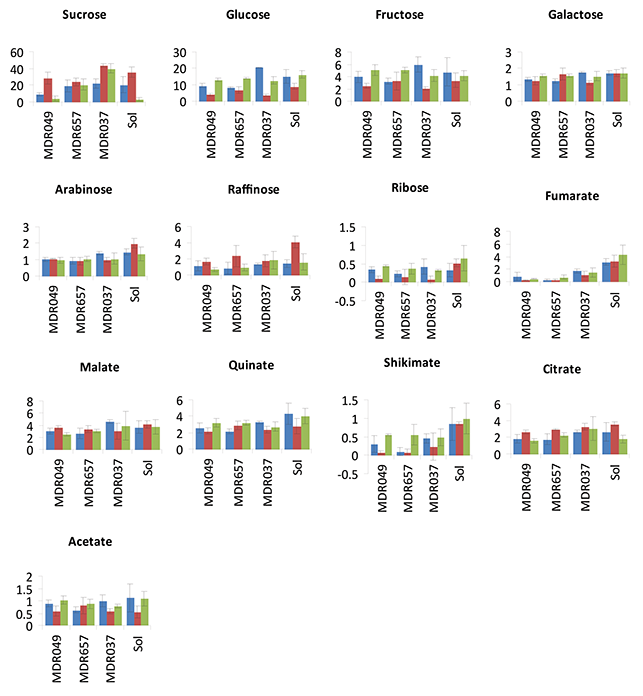

Supplement: Supplementary file 1 — Figure S1. Histograms showing metabolite concentrations (mg g−1 d.w.) of carbohydrate and organic acids in wheat leaf tissue, derived from 1D 1H nuclear magnetic resonance. Error bars show SD. Blue, control; red, aphid‐localised; green, systemic response. Note different scales on y‐axis. [file AAB-168-435-s001.tif]

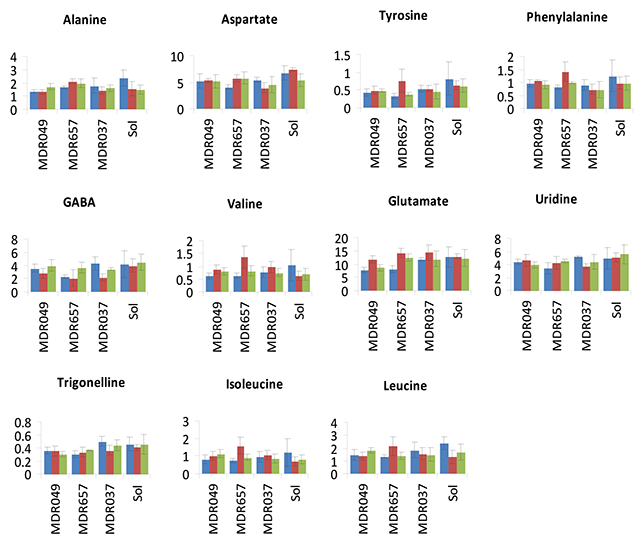

Supplement: Supplementary file 2 — Figure S2. Histograms showing concentrations (mg g‐1 d.w.) of amino acid, methyl donor and aromatic metabolites in wheat leaf tissue, derived from 1D 1H nuclear magnetic resonance. Error bars show SD. Blue, control; red, aphid‐localised; green, systemic response. Note different scales on y‐axis. [file AAB-168-435-s002.tif]
