# Supplementary material for: Triticum monococcum lines with distinct metabolic phenotypes and phloem‐based partial resistance to the bird cherry–oat aphid Rhopalosiphum padi
Source: Ann Appl Biol. 2016 Feb 29;168(3):435–49. doi: 10.1111/aab.12274 (PMC4982108; doi:10.1111/aab.12274)
Supplement: Supplementary file 3 — Table S1. Results from analysis of variance for metabolomic phenotyping of Triticum monococcum lines MDR049, MDR657, MDR037 and Triticum aestivum var. Solstice [file AAB-168-435-s003.docx]

|  | **Significance of differences (*P*-value) between treatments for each of the four lines** | | | | **Significance of differences (P-value) between lines for each of the treatments** | | |
| --- | --- | --- | --- | --- | --- | --- | --- |
| ***Metabolite*** | ***Plant line*** | | | | ***Treatment tissue*** | | |
|  | MDR049 | MDR657 | MDR037 | Solstice | Control | Localised | Systemic |
| Acetate | 0.637 | 0.092 | **0.043** | 0.222 | 0.355 | 0.452 | 0.307 |
| Alanine | 0.430 | 0.512 | 0.569 | 0.153 | 0.096 | 0.562 | 0.311 |
| Arabinose | 0.641 | 0.729 | 0.115 | 0.099 | **0.008** | **0.001** | 0.395 |
| Asparagine | 0.428 | 0.267 | 0.263 | 0.727 | **0.003** | **0.013** | **0.038** |
| Aspartate | 0.910 | **0.038** | 0.287 | 0.136 | 0.063 | **0.002** | 0.674 |
| Choline | **0.001** | **0.000** | **0.001** | 0.274 | 0.070 | **0.001** | 0.466 |
| Citrate | **0.026** | **0.036** | 0.752 | 0.059 | 0.293 | **0.017** | 0.187 |
| Fructose | 0.236 | **0.001** | **0.006** | 0.592 | 0.176 | 0.280 | 0.364 |
| Fumarate | 0.199 | 0.126 | 0.491 | 0.673 | 0.101 | **0.000** | **0.002** |
| GABA | 0.428 | **0.017** | **0.018** | 0.199 | 0.207 | 0.674 | 0.529 |
| Galactose | 0.414 | **0.064** | 0.307 | 0.460 | 0.199 | **0.043** | 0.363 |
| Glucose | 0.144 | **0.000** | **0.001** | 0.333 | **0.025** | 0.157 | 0.635 |
| Glutamate | **0.011** | **0.003** | 0.392 | 0.944 | 0.052 | 0.136 | 0.240 |
| Glutamine | **0.047** | **0.080** | 0.855 | 0.103 | 0.555 | 0.065 | **0.046** |
| Glycerol | **0.005** | **0.000** | **0.006** | 0.334 | 0.402 | **0.008** | 0.054 |
| Glycine Betaine | 0.969 | **0.030** | **0.003** | 0.333 | 0.214 | **0.001** | **0.000** |
| Guanosine | 0.062 | 0.418 | 0.434 | 0.608 | 0.425 | 0.294 | 0.122 |
| Isoleucine | 0.144 | 0.286 | 0.630 | 0.498 | 0.621 | 0.309 | 0.382 |
| Leucine | 0.567 | 0.584 | 0.728 | 0.441 | 0.459 | 0.776 | 0.713 |
| Malate | **0.017** | 0.497 | 0.483 | 0.818 | 0.051 | 0.349 | 0.584 |
| Octopamine | **0.030** | **0.004** | 0.806 | **0.016** | 0.055 | **0.000** | **0.009** |
| Phenylalanine | 0.164 | 0.148 | 0.632 | 0.659 | 0.449 | 0.149 | 0.499 |
| Quinate | 0.529 | **0.026** | 0.109 | 0.249 | **0.033** | 0.715 | 0.171 |
| Raffinose | 0.180 | 0.169 | 0.712 | **0.009** | 0.609 | **0.041** | 0.287 |
| Ribose | 0.052 | **0.018** | **0.044** | 0.307 | 0.496 | **0.001** | 0.236 |
| Shikimate | **0.013** | **0.030** | 0.495 | 0.855 | **0.045** | **0.003** | 0.167 |
| Succinate | **0.031** | **0.016** | 0.868 | 0.722 | 0.100 | 0.899 | 0.313 |
| Sucrose | 0.146 | 0.373 | 0.053 | 0.231 | 0.844 | 0.111 | **0.001** |
| Threonine | **0.001** | **0.001** | **0.026** | 0.103 | 0.118 | **0.008** | 0.431 |
| Trehalose | 0.092 | 0.067 | 0.058 | **0.002** | **0.019** | **0.027** | 0.443 |
| Trigonelline | 0.115 | 0.057 | 0.181 | 0.815 | **0.041** | 0.062 | 0.208 |
| Tyrosine | 0.213 | 0.243 | 0.765 | 0.676 | 0.175 | 0.897 | 0.324 |
| Uridine | **0.030** | 0.082 | 0.095 | 0.831 | 0.164 | **0.004** | 0.255 |
| Valine | 0.059 | 0.249 | 0.399 | 0.438 | 0.423 | 0.337 | 0.821 |
